# Exonuclease-assisted enrichment and base resolution analysis of pseudouridine in single-stranded RNA†

**DOI:** 10.1039/d4sc03576c

**Published:** 2024-10-21

**Authors:** Xin Fang, Ziang Lu, Yafen Wang, Ruiqi Zhao, Jing Mo, Wei Yang, Mei Sun, Xiang Zhou, Xiaocheng Weng

**Affiliations:** a College of Chemistry and Molecular Sciences, Key Laboratory of Biomedical Polymers-Ministry of Education, Wuhan University Wuhan Hubei 430072 P. R. China xcweng@whu.edu.cn; b School of Public Health, Wuhan University Wuhan Hubei 430071 P. R. China; c Department of Neurosurgery, Zhongnan Hospital of Wuhan University Wuhan Hubei 430071 P. R. China; d TaiKang Center for Life and Medical Sciences, Wuhan University Wuhan Hubei 430071 P. R. China; e Department of Otorhinolaryngology-Head and Neck Surgery, Zhongnan Hospital of Wuhan University Wuhan Hubei P. R. China

## Abstract

Pseudouridine (Ψ) is one of the most abundant RNA modifications, playing crucial roles in various biological processes. Identifying Ψ sites is vital for understanding their functions. In this study, we proposed a novel method for identifying Ψ sites with an improved signal-to-noise ratio. This method, called RNA exonuclease-assisted identification of pseudouridine sites (RIPS), combines specific CMC-labeling of Ψ sites with an exonuclease-assisted digestion strategy for the detection of Ψ sites. Utilizing exonuclease XRN1 to digest RNA strands not labeled by CMC, RIPS significantly reduces the background signal from unlabeled strands and enhances the positive signal of Ψ sites labeled by CMC, which terminates exonuclease digestion. As a result, we can enrich Ψ sites and identify them at single-base resolution. Considering the unique functions of single-stranded RNA (ssRNA), we employed RIPS to distinguish Ψ sites in single-stranded and double-stranded regions of RNA. Our results indicated that CMC could specifically label Ψ sites in ssRNA under natural conditions, enabling RIPS to selectively identify Ψ sites in ssRNA, which may facilitate the study on the functions of Ψ sites.

## Abstract 2

We developed RIPS for identification of Ψ sites with a higher signal-to-noise ratio by combining the CMC specific labeling and exonuclease digestion. What's more, we selectively identified Ψ sites in single-stranded RNA by applying RIPS.

## Graphical abstract

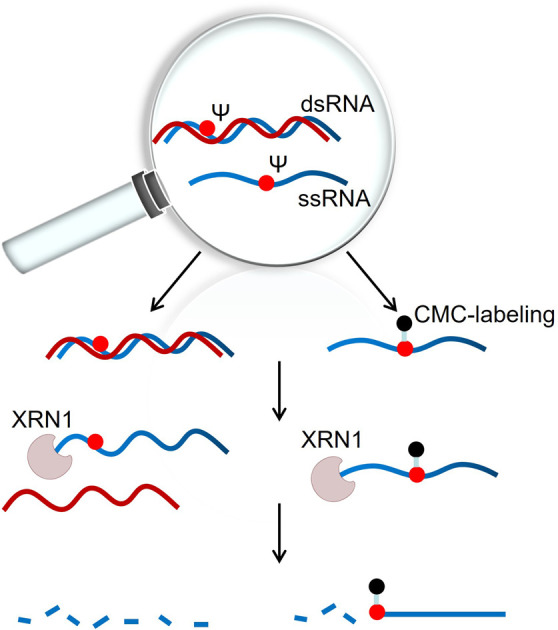

## Introduction

RNA plays important roles in various biological processes. In addition to the four common bases, researchers have discovered many modified bases that are present in RNA. To date, more than 160 post-transcriptional modifications have been identified in different types of RNA.^1–3^ Among these modifications, pseudouridine (Ψ) is one of the most prevalent modifications, found in rRNA, tRNA and even in mRNA, where it has essential functions.^4,5^ Many studies have demonstrated that Ψ can affect mRNA splicing, rRNA binding internal ribosome entry sites, translation fidelity, and RNA structure stability.^6–10^ More recently, research has demonstrated that Ψ in tRNA can predict leukemic progression in myelodysplastic syndrome.^11^

Ψ is an isomer of uridine (U) and is catalyzed by pseudouridine synthases (PUSs), which can be divided into two main groups: RNA-dependent box H/ACA ribonucleoproteins and RNA-independent PUSs.^12–15^ The base-pairing pattern of Ψ in RNA is similar to U, but it can form an additional hydrogen bond, giving it vital roles in regulating the RNA structure.^9,16,17^ It's well-known that the RNA secondary structure is an important regulatory element for post-transcriptional gene expression,^18–20^ and several methods have been developed to map the RNA secondary structure, such as Keth-seq,^21^ DMS-seq,^22^ icSHAPE,^23^ and EDC or CMC based probing technology.^24^ It has been reported that the RNA structure could impact RNA modification,^25^ while RNA modification can also regulate the RNA structure.^26^ Ψ located in a duplex could stabilize RNA folding domains,^27^ while Ψ sites in ssRNA, such as the loop of the hairpin, play special roles in the translation,^28^ splicing,^29^ and immune response.^30^ Previous studies have revealed that three Ψ modifications (Ψ1911, Ψ1915, and Ψ1917) in the 23S rRNA helix 69 could modulate conformation and structural dynamics of the helix in different ways, and the Ψ1917C mutation may alter the RNA structure and become one of the important reasons for inhibiting the translation process in *E. coli*.^31,32^ Therefore, distinguishing Ψ sites in ssRNA and double-stranded RNA (dsRNA) will facilitate research on the function of Ψ.

Various research tools and techniques have been developed to map Ψ sites, which included Ψ-seq, PSI-seq, Pseudo-seq, HydraPsiSeq, CeU-seq, BID-seq, PRAISE and the nanopore-based method.^4,33–40^ The classical method for Ψ profiling is a chemically assisted method using *N*-cyclohexyl-*N*′-(2-morpholinoethyl)-carbodiimide metho-*p*-toluenesulfonate (CMC) to map transcriptome-wide Ψ sites.^41^ CMC can react with guanosine (G), U and Ψ to produce the corresponding adducts, G-CMC, U-CMC and Ψ-CMC. However, G-CMC and U-CMC adducts undergo CMC removal under alkaline treatment, while Ψ-CMC does not because it is more stable. The Ψ-CMC blocks the reverse transcriptase and prevents reverse transcription at one nucleotide 3′ to the Ψ-CMC sites, enabling single-base resolution mapping of Ψ. Although this method is commonly used, it has certain limitations. It doesn't pre-enrich Ψ-containing RNA, so lower abundance Ψ sites may be missed due to the high background caused by a large number of RNA fragments without CMC labeling. N_3_-CMC, a derivative of CMC, was developed to identify Ψ in RNA with higher accuracy and sensitivity, using biotin to enrich RNA containing Ψ (CeU-seq).^36^ However, the synthesis and storage of N_3_-CMC are challenging, limiting its widespread application. Recently, bisulfite-induced deletion sequencing (BID-seq) and quantitative Ψ assessment *via* bisulfite/sulfite treatment (PRAISE) were reported, which use a bisulfite-mediated reaction to quantitatively convert Ψ to a sulfite adduct of Ψ without cytidine deamination, resulting in a unique deletion signature at the Ψ sites during reverse transcription.^37,38,42,43^ However, sufficient read coverage is required to identify low-abundance Ψ sites because of the absence of enrichment methods.

Herein, an RNA exonuclease-assisted identification of pseudouridine sites (RIPS) was developed to address the issue of the high background caused by RNA fragments not labeled by CMC. This method involved using CMC to specifically label Ψ.^44,45^ XRN1 is a 5′ exonuclease that could degrade RNA starting from its 5′ end. Due to CMC steric hindrance, XRN1 digestion is terminated at one nucleotide 5′ to the Ψ-CMC sites in CMC-labeled strands. As a result, RIPS can enrich Ψ-containing RNA strands labeled by CMC and identify Ψ sites with a higher signal-to-noise ratio. It is worth noting that Ψ within different RNA secondary structures may have distinct biological roles, but has not received sufficient attention. The RIPS method can distinguish Ψ in ssRNA or dsRNA because CMC can only label Ψ in ssRNA, not in dsRNA, which may provide new insights into the understanding of functions of Ψ modifications.

## Results and discussion

### Development of the RIPS strategy for Ψ identification

**Fig. 1:**
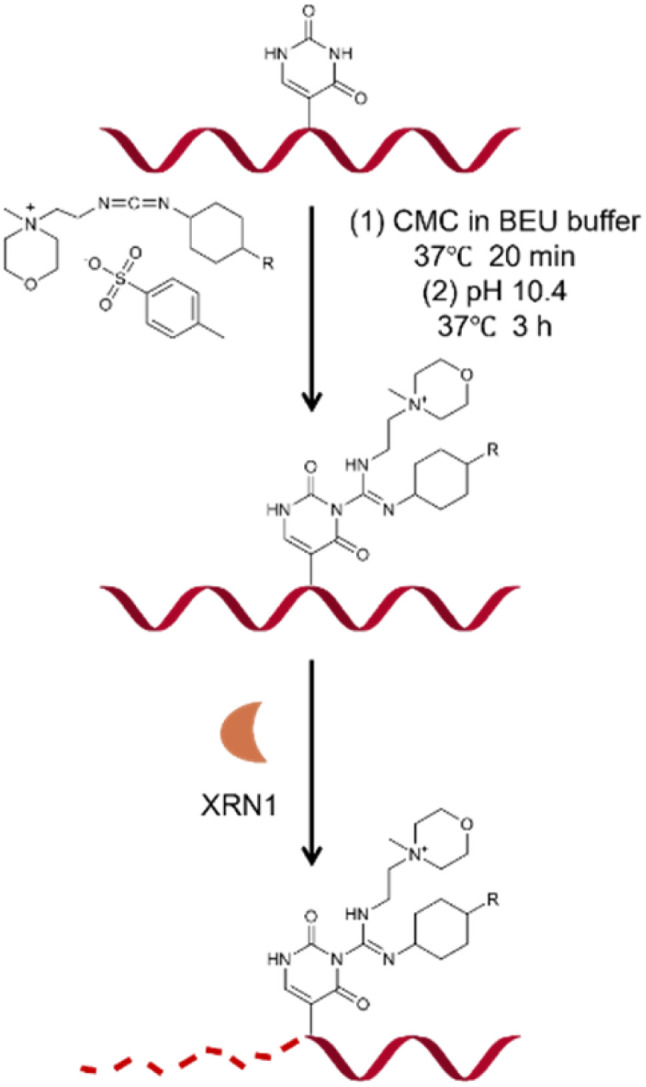
Schematic illustrating the principle of CMC labeling and XRN1 exonuclease digestion.

**Fig. 2:**
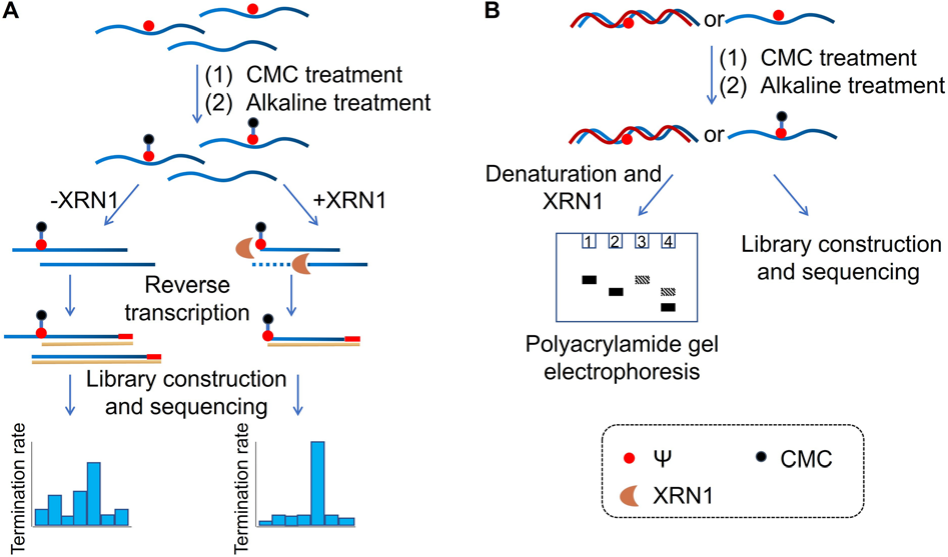
Schematic of the RIPS strategy. (A) CMC was used to specifically label Ψ sites contained in RNA. And unlabeled RNA was digested by XRN1 exonuclease from the 5′ end to the 3′ end, while the Ψ-CMC adduct caused the digestion to terminate before Ψ sites of labeled RNA, thereby enabling Ψ detection with a higher signal-to-noise ratio. (B) RIPS strategy for the distinction of Ψ sites in single-stranded and double-stranded regions of RNA. CMC could selectively label Ψ sites in ssRNA under natural conditions, while dsRNA failed to be labeled. Selective labeling of Ψ was confirmed by gel electrophoresis analysis. And the Ψ sites in ssRNA were specifically identified by high-throughput sequencing.

Based on previous studies, we have developed a novel method for Ψ detection called RIPS. This method employed exonuclease to digest RNA lacking Ψ modifications that cannot be labeled by CMC. Meanwhile, CMC-labeling at the Ψ sites in Ψ-containing RNA hindered exonuclease digestion and made the digestion terminate before Ψ sites due to the steric hindrance of CMC (Fig. 1). Therefore, a large number of RNA strands that weren't labeled by CMC could be excluded from library construction and sequencing, achieving the desired enrichment effect (Fig. 2A). Furthermore, as CMC can only label Ψ sites in ssRNA but not dsRNA, RIPS can selectively identify Ψ in ssRNA with a higher signal-to-noise ratio by digesting unlabeled dsRNA (Fig. 2B).

### The feasibility verification of the RIPS strategy

**Fig. 3:**
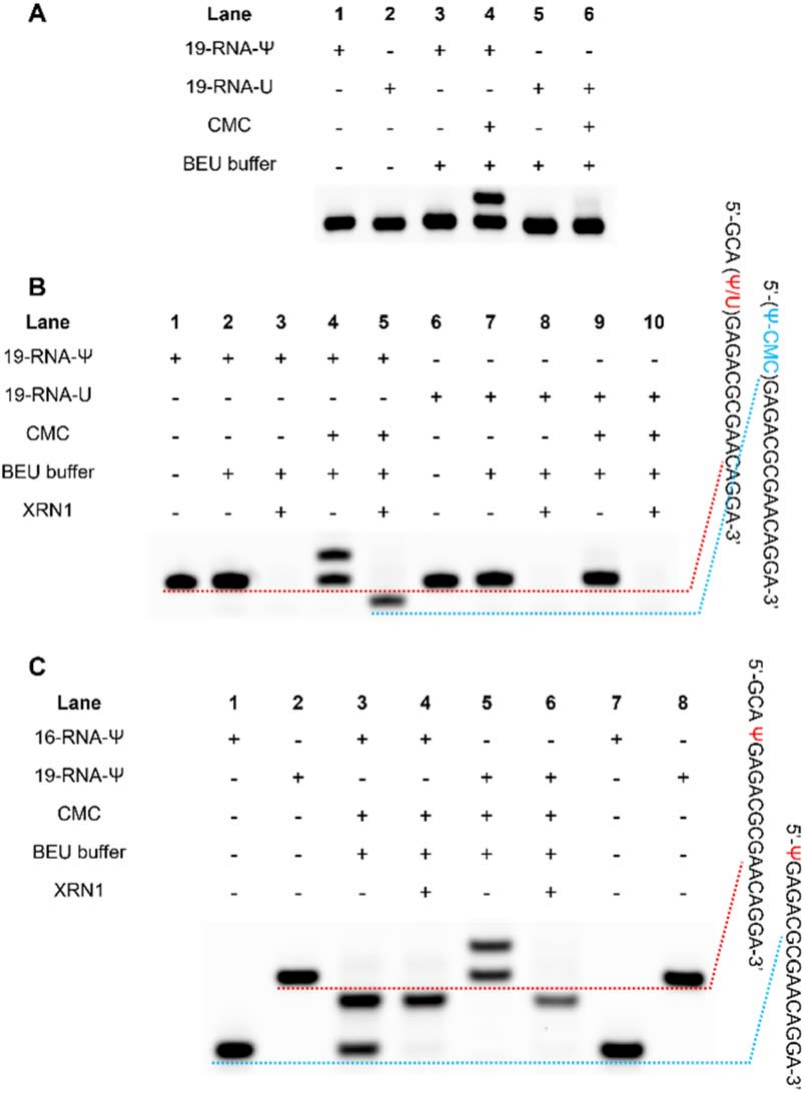
Preliminary experimental validation of RIPS. (A) RNAs containing Ψ or U were treated with CMC, and separated by gel electrophoresis. (B) Preliminary validation of the feasibility of the RIPS strategy. The RNA containing Ψ or U was reacted with CMC. XRN1 was used to digest unlabeled RNA. (C) Gel electrophoresis analysis of the products of 16-RNA-Ψ and 19-RNA-Ψ reacted with CMC and treated with XRN1.

Initially, two 19-nucleotide (nt) RNAs containing a Ψ site (19-RNA-Ψ) or a U site (19-RNA-U) were incubated with CMC, respectively. Using denaturing polyacrylamide gel electrophoresis, we observed that 19-RNA-Ψ migrated slower after reacting with CMC and undergoing alkaline treatment, while 19-RNA-U did not (Fig. 3A). High-resolution mass spectrometry confirmed that CMC could indeed label the Ψ-containing RNA (Fig. S1†). We also optimized the conditions for the reaction of the RNA with CMC (Fig. S2†).

Subsequently we treated the above 19 nt RNA with XRN1 exonuclease after CMC labeling. Denaturing polyacrylamide gel electrophoresis revealed the emergence of a shorter RNA strand in the 19-RNA-Ψ sample treated with CMC and XRN1, which was expected to result from the termination of digestion before the Ψ sites. In contrast, the 19-RNA-U sample treated with CMC and XRN1 displayed a reduced amount of the original RNA strand (Fig. 3B).

To confirm whether exonuclease cleavage terminated before the Ψ site, a 16 nt RNA strand (16-RNA-Ψ) was synthesized, with the three bases in front of the Ψ site of the 19-RNA-Ψ removed. CMC labeling and exonuclease cleavage were conducted on both 16-RNA-Ψ and 19-RNA-Ψ. Gel electrophoresis analysis showed that the products in Lanes 3, 4, and 6 were identical, though Lane 3 still exhibited some residual RNA material (Fig. 3C). To further verify the products, mass spectrometry was conducted. The results revealed that the molecular weights of both 16-RNA-Ψ and 19-RNA-Ψ treated with CMC labeling and XRN1 digestion were 6107.9, matching the molecular weight of 16-RNA-Ψ (5855.7) plus the molecular weight of one CMC molecule (252.2) (Fig. S3†). These findings provided compelling evidence that XRN1 digestion indeed terminated at the Ψ site of 19-RNA-Ψ following CMC labeling and exonuclease treatment.

These results demonstrated that CMC effectively impeded exonuclease digestion from the 5′ end by its steric hindrance, causing the digestion to terminate before the CMC-labeled Ψ sites and producing a shorter band, while the U-containing RNA didn't.

### Optimization of experimental conditions for the RIPS strategy

We further optimized the digestion conditions to ensure that CMC-unlabeled RNA strands were almost completely digested, while the CMC-labeled RNA strands could hinder digestion before the Ψ sites as much as possible. First, we optimized the concentration of exonuclease (0, 0.125, 0.25, 0.3, 0.35, 0.4, 0.45, 0.5, 1, and 2 U). Gel electrophoresis was used to assess the digestion efficiency of XRN1, with the best efficiency observed at 0.25 U exonuclease (Fig. S4†). Next, we optimized the digestion time of exonuclease (0, 5, 10, 15, 30, 40, 50, and 60 min). The optimal digestion conditions for 19-RNA-Ψ were determined to be 100 ng of RNA, NEB buffer 3, 0.5 U enzyme concentration, and 5 minutes of digestion time (Fig. S4†). These experimental results demonstrated that the combination of CMC-specific labeling technology and the exonuclease digestion method provided an efficient strategy to enrich Ψ-containing RNA which was labeled by using CMC.

**Fig. 4:**
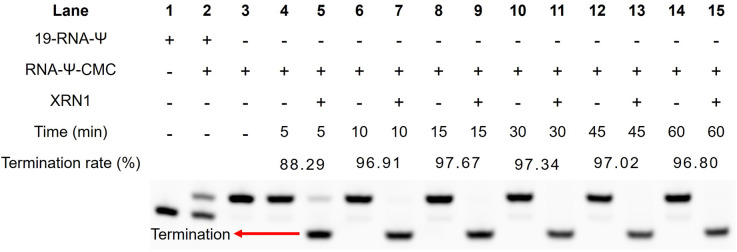
Termination rate of XRN1 digestion influenced by the Ψ-CMC adduct. The CMC-labeled RNA was separated by HPLC and subjected to XRN1 digestion. RNA was separated by gel electrophoresis and the termination rate was calculated by comparing the signals of shortened RNA and untreated RNA.

Subsequently, we assessed the termination efficiency of XRN1 digestion for CMC-labeled RNA. CMC-labeled RNA was separated from other unlabeled RNA by HPLC (Fig. S5†). The recovered CMC-labeled RNA strand (RNA-Ψ-CMC) was digested by XRN1 with a time gradient (0, 5, 10, 15, 30, 45, and 60 min). Compared to the control group, which did not undergo digestion, the CMC-labeled RNA strand demonstrated a 95% probability of impeding exonuclease cleavage after 10 minutes (Fig. 4), indicating that the enzymatic digestion strategy enriched CMC-labeled RNA and enabled high-sensitivity detection of Ψ.

### Ψ identification by the RIPS strategy using high-throughput sequencing

**Fig. 5:**
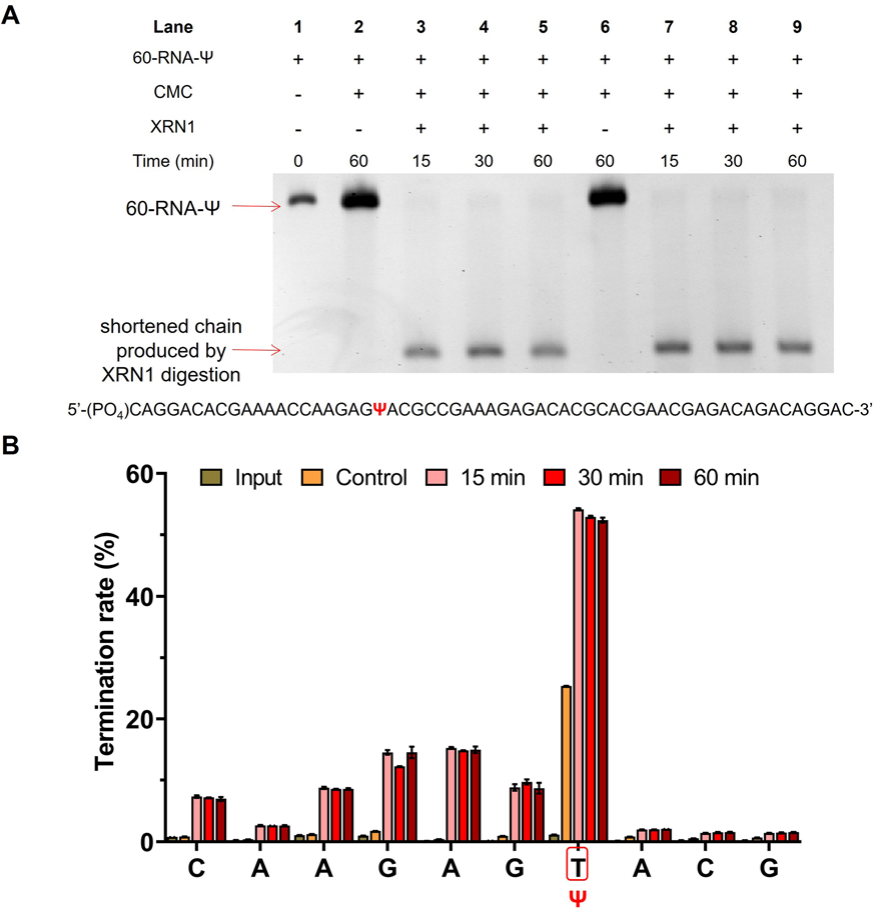
RIPS strategy identified Ψ sites with a higher signal-noise ratio. (A) Gel electrophoresis analysis exhibited the efficiency of XRN1 digestion toward 60-RNA-Ψ. (B) Validation of the RIPS strategy for Ψ site identification of 60-RNA-Ψ by high-throughput sequencing. RNA was treated with XRN1 for different times (0, 15, 30, and 60 min). Input: 60-RNA-Ψ input. Control: 60-RNA-Ψ treated with CMC only. 15 min: 60-RNA-Ψ treated with CMC and XRN1 for 15 min. 30 min: 60-RNA-Ψ treated with CMC and XRN1 for 30 min. 60 min: 60-RNA-Ψ treated with CMC and XRN1 for 60 min. The position of Ψ was marked with a red frame (*n* = 2 biologically independent experiments).

In order to verify that the exonuclease digestion does stop before the Ψ sites, and that the method could indeed be used for the identification of the Ψ sites with a higher signal-to-noise ratio, we used a longer 60 nt RNA containing a Ψ site (60-RNA-Ψ) to assess CMC labeling efficiency and exonuclease digestion efficiency through library sequencing analysis. The samples treated with CMC were subjected to an exonuclease digestion time gradient (15, 30, and 60 min). The gel electrophoresis analysis showed that a shortened chain was produced by XRN1 digestion as previously described (Fig. 5A). The sequencing results demonstrated that obvious reverse transcription termination occurred at one nucleoside 3′ to the Ψ sites in the samples treated with CMC, with a termination rate of less than 30%. Meanwhile, the samples treated with both CMC and XRN1 were found to indeed undergo reverse transcription termination before the Ψ sites, with a higher termination efficiency compared to CMC-only treated samples. The reverse transcription termination efficiency of CMC and XRN1 treated samples exceeded 55% (Fig. 5B and Dataset S1†). This evidence supported the notion that the exonuclease could digest CMC-unlabeled RNA within 15 min of digestion time, and that CMC-labeling in RNA could terminate digestion of XRN1 in front of the Ψ sites, which could thereby improve the reverse transcription termination rate of the Ψ site and enhance the signal-to-noise ratio. The results indicated the feasibility and reliability of the RIPS method.

### RIPS strategy identifies Ψ sites in total RNA

**Fig. 6:**
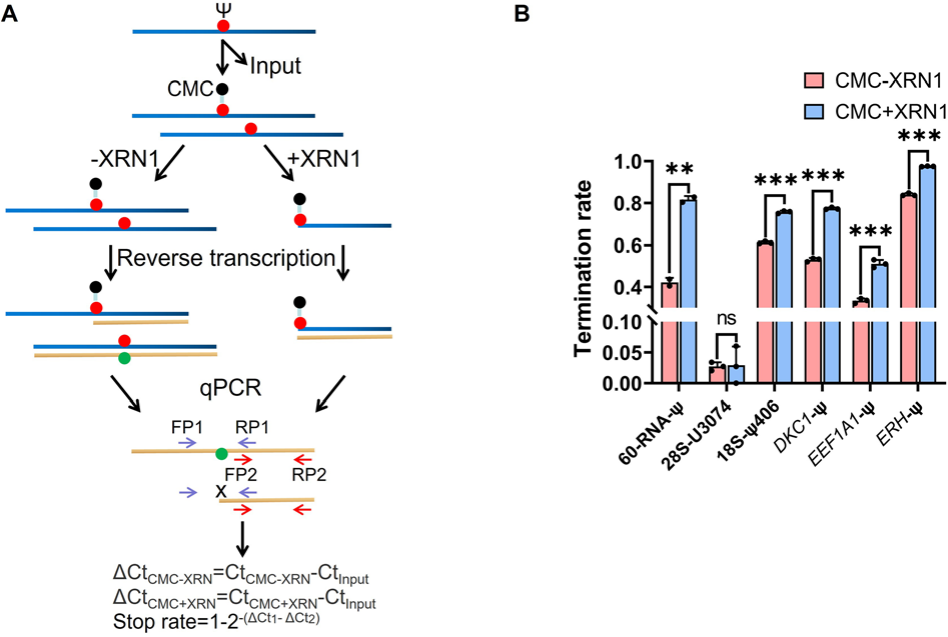
RIPS strategy identified Ψ sites in total RNA with a higher signal-to-noise ratio. (A) Schematic for the calculation of the termination rate of Ψ sites by qPCR. Two pairs of primers were designed (FP1 and RP1, and FP2 and RP2). Using Input as the control, the termination rate was calculated using 1–2^−(ΔCt_1_−ΔCt_2_)^. ΔCt_1_ was defined as the relative content of the treated samples when amplified with FP1 and RP1 primers. ΔCt_2_ was defined as the relative content of the treated samples when amplified with FP2 and RP2. (B) Termination rate of 60-RNA-Ψ and several known sites in rRNA and even mRNA using RIPS or only CMC labeling. The specific sites are listed in Table S3.† Error bars indicate mean ± s.d. for 3 technical replicates.

Subsequently, we sought to detect several Ψ sites located in rRNA and even mRNA using RIPS (Table S3†). We used real-time quantitative PCR (qPCR) to quantify the termination rate, designing two pairs of primers, with one pair of primers (FP1 and RP1) located on either side of the Ψ site and the other pair of primers (FP2 and RP2) located downstream of the Ψ site (Table S2†). As shown in Fig. 6A, only cDNAs without reverse transcription termination were successfully amplified by FP1 and RP1, while cDNAs both with or without reverse transcription termination could be successfully amplified by FP2 and RP2. We used Input as the control and divided the relative content of FP1 and RP1 amplification by the relative content of FP2 and RP2 amplification (2^−(ΔCt_1_−ΔCt_2_)^). The termination rate was calculated using 1–2^−(ΔCt1−ΔCt_2_)^. The previous conditions for Ψ detection on RNA from HEK293T cells resulted in incomplete digestion, likely due to the more complex structure of the samples and the broader variety of RNA species. To address this, we re-optimized the experimental conditions for these complex RNA samples. The optimal XRN1 digestion conditions were determined to be 100 ng of RNA, NEB buffer 1, 1 U enzyme concentration, and 5 minutes of digestion time (Fig. S6†). Initially, we digested the input samples without CMC labeling to eliminate the potential influence of the RNA structure and other variables on the exonuclease digestion process (Fig. S7†). The results showed that most of the unlabeled RNA was effectively digested. Subsequently, we successfully identified several known Ψ sites in rRNA and mRNA with a higher signal-to-noise ratio (Fig. 6B).^36,37^ Furthermore, our analysis of the termination rate at 28S-U3074 revealed negligible differences with or without XRN1 treatment, supporting the efficacy of RIPS in detecting Ψ sites in genuine samples while enhancing the signal clarity.

### RIPS strategy enables selective identification of Ψ sites in single-stranded RNA

**Fig. 7:**
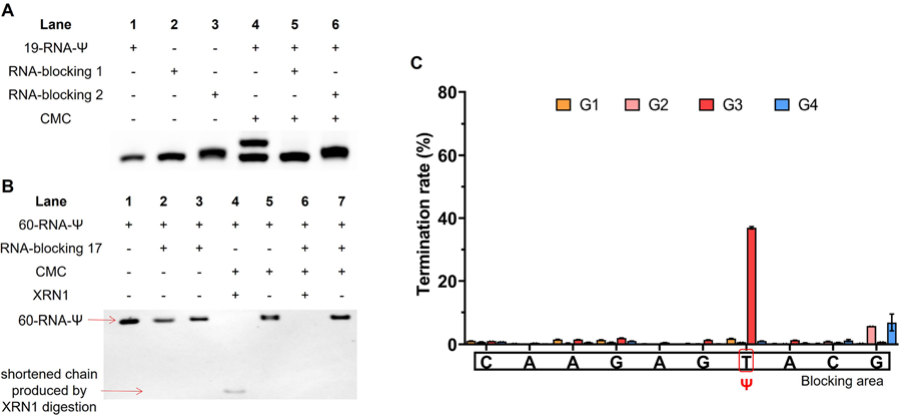
Distinguishing of Ψ sites in single-stranded and double-stranded regions of RNA by the RIPS strategy. (A) RNA-blocking 1 or 2 was annealed with the 19 nt RNA to form dsRNA, allowing the Ψ sites to be blocked in the double-stranded structure, and the blocked or unblocked oligonucleotide chain was incubated with CMC under natural conditions. (B) RNA-blocking 17 was annealed with the 60 nt RNA and treated with CMC, the same as (A). After denaturation treatment, RNA was further treated with XRN1. (C) High-throughput sequencing validated that CMC only labeled Ψ sites in single-stranded regions of RNA. G1: single-stranded 60-RNA-Ψ input. G2: double-stranded 60-RNA-Ψ input. G3: single-stranded 60-RNA-Ψ treated with CMC. G4: double-stranded 60-RNA-Ψ treated with CMC. The position of Ψ was marked with a red frame and the partial blocking area was marked with a black square (*n* = 2 biologically independent experiments).

In addition, we also conducted a preliminary study of Ψ sites in different structural regions of RNA. RNA has a complex secondary or tertiary structure, and Ψ sites located in various RNA structural regions may possess different biological functions. In ssRNA, the hydrogen atoms at the N3 position of Ψ bases don't participate in base-pairing, allowing CMC to specifically label Ψ. However, when the Ψ sites are located in dsRNA, the hydrogen atoms at the N3 position would be involved in hydrogen bond formation, and thus prevent CMC from labelling the Ψ sites. To examine the labeling efficiency of CMC to ssRNA and dsRNA, we designed two partly complementary strands to block the Ψ sites in 19-RNA-Ψ. The 19-RNA-Ψ was annealed with the two complementary strands to form dsRNA, in which the Ψ sites were blocked in the double-stranded regions (Fig. S8†). The single-stranded 19-RNA-Ψ and dsRNA were both treated with CMC and analyzed by denaturing polyacrylamide gel electrophoresis. During the subsequent denaturing polyacrylamide gel electrophoresis, the RNA secondary structure was opened, and it could be seen that only the 19-RNA-Ψ that was treated with CMC showed two electrophoretic bands, while the blocking-RNA that was treated with CMC displayed only one band (Fig. 7A), indicating that only the Ψ in the single-stranded regions could be labeled by CMC.

We then selected the previously used 60-RNA-Ψ and designed a series of partly complementary RNA to perform XRN1 digestion, library construction and sequencing. After annealing, the Ψ sites were blocked in the double-stranded regions. We finally selected the RNA-blocking 17, as it had best blocking efficiency (Fig. S9†). The single-stranded 60-RNA-Ψ and double-stranded RNA were treated with CMC. Then both RNAs were denatured and treated with XRN1. We found that the 60-RNA-Ψ in dsRNA was completely digested by XRN1, because the Ψ-containing RNA failed to be labeled by CMC. However, the single-stranded 60-RNA-Ψ was only partly digested by XRN1 and displayed a shortened chain, suggesting that the digestion was resisted by CMC labeling (Fig. 7B). This result further demonstrated that only Ψ in the single-stranded regions could be specifically labeled by CMC, while Ψ in the double-stranded regions could not. We further performed additional library construction and sequencing analysis of Ψ in the ssRNA or dsRNA under CMC labeling. The results showed that reverse transcription termination was apparent at Ψ sites in single-stranded regions but not in double-stranded regions (Fig. 7C and Dataset S2†), proving that RIPS could be used to distinguish Ψ sites located at different RNA secondary structures.

**Fig. 8:**
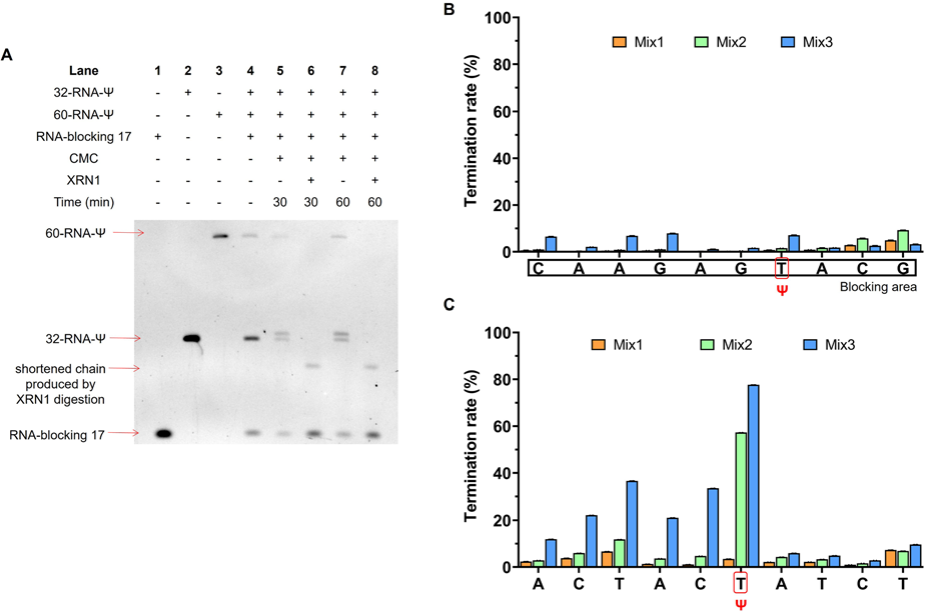
RIPS strategy selectively identified Ψ sites in ssRNA from a mixture of ssRNA and dsRNA. (A) Single-stranded 32-RNA-Ψ and double-stranded 60-RNA-Ψ were mixed and treated with CMC under natural conditions. After that, the mixture was further treated with XRN1 under denaturing conditions. RNAs were analyzed by gel electrophoresis. (B) Reverse transcription termination rate of bases near the modified base of 60-RNA-Ψ in the mixture was analyzed by sequencing. (C) Reverse transcription termination rate of bases near the modified base of 32-RNA-Ψ in the mixture was analyzed by sequencing. Mix1: single-stranded 32-RNA-Ψ and double-stranded 60-RNA-Ψ mixture input. Mix2: single-stranded 32-RNA-Ψ and double-stranded 60-RNA-Ψ mixture treated with CMC only. Mix3: single-stranded 32-RNA-Ψ and double-stranded 60-RNA-Ψ mixture treated with CMC and XRN1. The position of Ψ was marked with a red frame and the partial blocking area was marked with a black square (*n* = 2 biologically independent experiments).

Finally, we designed another ssRNA (32-RNA-Ψ) containing a Ψ and mixed it with the double-stranded 60-RNA-Ψ, to examine whether our method could selectively identify Ψ sites in ssRNA from a mixture of ssRNA and dsRNA. The mixture was incubated with CMC and treated with XRN1. Denaturing polyacrylamide gel electrophoresis revealed four bands following CMC treatment, suggesting that single-stranded 32-RNA-Ψ was specifically labeled by CMC (Fig. 8A). After introducing XRN1, a shortened band appeared and the top band disappeared, indicating that 32-RNA-Ψ was successfully labeled by CMC and the Ψ-CMC adduct successfully resisted XRN1 digestion, while the double-stranded 60-RNA-Ψ failed to be labeled and was almost completely digested by XRN1. RNA-blocking 17 remained undigested by XRN1 due to the absence of phosphate modification at its 5′ end which prevented XRN1 from digesting this RNA strand. Subsequently, we performed library construction and sequencing analysis of these sets of sample Lanes 4, 5, 6, 7 and 8. By analyzing the sequencing results of 60-RNA-Ψ, we found a low reverse transcription termination rate of modification sites under CMC treatment, suggesting that it failed to be labeled by CMC (Fig. 8B). In contrast, the sequencing results of 32-RNA-Ψ exhibited a high reverse transcription termination rate of Ψ in treated samples, and XRN1 treatment indeed improved the termination rate of Ψ (Fig. 8C and Dataset S3†). These results further confirmed that CMC can indeed be used to distinguish Ψ in single- and double-stranded structural regions. Our exonuclease-assisted method significantly improved the accuracy and sensitivity of Ψ identification. We believe that our preliminary results will contribute to the subsequent studies about identifying Ψ sites in different structural regions of RNA.

## Conclusions

In conclusion, we established a new Ψ identification method, a chemical and exonuclease-assisted method (RIPS). This method employs CMC to specifically label Ψ sites, followed by exonuclease digestion. This strategy efficiently enriches Ψ, enhancing the signal-to-noise ratio and detection sensitivity. Our results demonstrated that XRN1 digestion could efficiently improve the reverse transcription termination rate of CMC-labeled RNA, reducing the background signal and enhance the positive signal, which indicated that RIPS is an efficient and meaningful technique for the identification of Ψ. In addition, RIPS can distinguish Ψ located in ssRNA and dsRNA. It is believed that RIPS is also a way of thought for future studies on Ψ mapping across different structural regions of RNA. Moreover, RIPS can provide an effective idea for the accurate mapping of Ψ, which can contribute to our understanding of Ψ functions in biology and medicine.

## Author contributions

X. F., and X. W. conceived the idea for this study. X. F. designed the experiments. X. F., R. Z., and M. S. verified and developed the method, and performed all the experiments. Z. L. designed and performed the bioinformatics analysis. Y. W., J. M., and W. Y. contributed to the design of the experiments. X. F., and X. W. wrote the manuscript with help from all authors.

## Conflicts of interest

There are no conflicts to declare.

## Supplementary Material

SC-015-D4SC03576C-s001

SC-015-D4SC03576C-s002

SC-015-D4SC03576C-s003

SC-015-D4SC03576C-s004

## Data Availability

Sequencing data have been deposited into the Gene Expression Omnibus (GEO). The accession number is GSE229317. To review GEO accession GSE229317, go to https://www.ncbi.nlm.nih.gov/geo/query/acc.cgi?acc=GSE229317. Enter token enejysgyrfcpdef into the box.
