# Supplementary material for: Exonuclease-assisted enrichment and base resolution analysis of pseudouridine in single-stranded RNA
Source: Chem Sci. 2024 Oct 21;15(45):19022–8. doi: 10.1039/d4sc03576c (PMC11515940; doi:10.1039/d4sc03576c)
Supplement: SC-015-D4SC03576C-s004 [file SC-015-D4SC03576C-s004.pdf]

Supplementary Information (SI) for Chemical Science.
This journal is © The Royal Society of Chemistry 2024

Supporting Information

Exonuclease-assisted enrichment and base resolution analysis of pseudouridine in single-stranded RNA

Xin Fang,[‡a] Ziang Lu,[‡a] Yafen Wang,[‡b] Ruiqi Zhao,[‡c] Jing Mo,[a] Wei Yang,[a] Mei Sun,[a] Xiang Zhou[a, d] and Xiaocheng Weng[* a, e]

‡ These authors contributed equally to this work.

Table of Contents

Materials **2**
Methods **2**
CMC specifically labeled Ψ 2
Time and concentration selection for CMC specifically labeled Ψ 2
Identification of Ψ and Ψ-CMC adduct by HPLC-MS/MS 2
XRN1 exonuclease treatment 2
Optimization for XRN1 exonuclease treatment 2
ESI-MS analysis 3
Products separation by HPLC and stop rate of XRN1 digestion 3
Library preparation 3
Data analysis 4
Cell culture and RNA extraction 4
Ψ detection in total RNA using RIPS 4
Ψ distinction in RNA single-stranded and double-stranded region 4
Supplementary Tables and Figures **5**
Reference **14**

Materials

RNA used in this work were purchased from Takara, the sequences were listed in Table S1. DNA primers used in this work were purchased from Genecreate, the sequences were listed in Table S2. The specific sites of total RNA detected in this experiment were listed in Table S3.

Methods

CMC specifically labeled Ψ

To test the reaction specificity of CMC (Sigma) and pseudouridine (Ψ), synthetic FAM labeled RNA was used in this study: 16-RNA-Ψ, 19-RNA-Ψ and 19-RNA-U. Their sequences were listed in Table S1. These oligos were reacted with 0.2 M CMC in BEU buffer (7 M urea, 4 mM EDTA pH 8.0, 50 mM Bicine pH 8.5) at 37°C for 30 min followed by purification with ethanol precipitation.[1, 2] Control samples were treated with BEU buffer only. Purified RNA and sodium carbonate buffer (100 mM Na_2CO_3 pH 10.4) were mixed in a 1:1 ratio and incubated at 37°C for 3 h to remove CMC from U and G residues. RNA was purified by ethanol precipitation. The products were separated by denaturing polyacrylamide gel electrophoresis (urea-PAGE), and visualized by the fluorecence of FAM using Molecular Imager® ChemiDocTM XRS+ Imaging System (Bio-Rad).

Time and concentration selection for CMC specifically labeled

19-RNA-Ψ was reacted with 0, 20, 40, 60 μL 0.2 M CMC in BEU buffer at 37°C for 30 min. The following operations were same as previous description and the products were analyzed on a 20% urea-PAGE. We calculated the relative reaction efficiency through the blackness of the gel bands. Next, 19-RNA-Ψ was reacted with 40 μL 0.2 M CMC in BEU buffer at 37°C for 0, 10, 15, 20, 30 min, and further treated as the same way.

Identification of Ψ and Ψ-CMC adduct by HPLC-MS/MS

The oligos and CMC reactions were carried out as previously described. After purified by ethanol precipitation, we treated the products at 37°C for 2 h, with 1 μL Nuclease P1 (1 U/μL, NEB), 5 μL 5×Reaction buffer (10 mM $ZnCl_2$, 100 mM NaCl), and 19 μL RNase-free H_2O. Then 2.5 μL 1 M NH_4HCO_3 and 1 μL rSAP (1 U/μL, NEB) were added. The mixture was incubated at 37°C for another 2 h. The mixture was centrifuged at 17,000 g at room temperature for 15 min and the supernatant was collected to a new tube. 10 μL of the solution was loaded to HPLC-MS/MS for analysis.

XRN1 exonuclease treatment

The experimental operations of CMC and Ψ reaction were the same as before. After alkaline treatment and purification by ethanol precipitation, the product RNA was subjected to 5' to 3' exonuclease treatment with 0.5 μL XRN1 (1 U/μL, NEB) in NEB buffer 3 and 1 μL Ribolock RNase Inhibitor (40 U/μL, Thermo scientific), which was shook at 1000 rpm at 37℃. XRN1 was inactivated by heating at 70℃ for 10 min.[3] The products were analyzed on a 20% urea-PAGE.

Optimization for XRN1 exonuclease treatment

The experimental steps were the same as before. First, we optimized the exonuclease concentration gradient. 0, 1/16, 1/4, 1/2, 1, 2 μL XRN1 (1 U/μL, NEB) was added and the samples were shaken at 1000 rpm for 30 min at 37°C. Then, 0, 0.25, 0.3, 0.35, 0.4, 0.45, 0.5 μL XRN1 was carried out as described previously. Next, we optimized XRN1 digestion time. We performed enzyme digestion time gradient experiments using 0.25 U and 0.5 U exonuclease respectively, and selected 5, 10, 15, 30, 40, 50, 60 min enzyme digestion time. The parallel operation without XRN1 was used as control. For samples with a simple structure and low RNA complexity, such as synthetic RNA, the

optimal digestion conditions were determined to be 100 ng of RNA, NEB buffer 3, 0.5 U enzyme concentration, and 5 min digestion time. For samples with more complex RNA secondary structures and a greater diversity of RNA, the enzyme digestion conditions were established as 100 ng of RNA, NEB buffer 1, 1 U enzyme concentration, and 5 min digestion time.

ESI-MS analysis

16-RNA-Ψ and 19-RNA-Ψ were treated with CMC and XRN1 as above. The products were analyzed by ESI-MS. The ESI-MS recorded the signals using negative reflector mode.

Products separation by HPLC and stop rate of XRN1 digestion

19-RNA-Ψ raw material, and the products after reaction with CMC were passed into Dionex Ultimate 3000 hybrid LTQ Orbitrap Elite Velos Pro (Thermo Scientific) with a Thermo Scientifific Hypersil ODS Column (250 mm ×4.6 mm, 5 µm, C18). The gradient of elution buffer for LC is shown below (solvent A: TEAA buffer pH 7.0; solvent B: acetonitrile):

Time (min)	Flow (mL min^{-1})	%A	%B
0	0.2	95	5
5	0.2	95	5
40	0.2	60	40
45	0.2	0	100
50	0.2	0	100
51	0.2	95	5

Then the samples with different retention time were recovered separately, and the recovered samples were analyzed by denaturing polyacrylamide gel electrophoresis. The recovered CMC-specific labeled RNA strand (RNA-Ψ-CMC) was digested by exonuclease for an exonuclease digestion time gradient (0, 5, 10, 15, 30, 45, 60 min) with fixed 0.25 U enzyme concentration.

Library preparation

10 µL RNA was mixed with 1 µL 2 µM specific RT primer (Table S2), and denatured at 80°C for 2 min and then chilled on ice immediately. After annealing procedure, 4 µL 5×First strand buffer (NEB), 0.6 µL 100 mM $MgCl_2$, 2 µL 10 mM dNTPs mix (dATP, dCTP, dGTP and dTTP, NEB), 1 µL Ribolock RNase Inhibitor (40 U/µL, Thermo scientific), 1 µL 0.1 M DTT and 1 µL Superscript III reverse transcriptase (200 U/ µL, Thermo scientific) were added into the RNA-primer mix, followed by incubation at 25°C for 3 min，42°C for 7 min，52°C for 30 min and hold at 4°C. Then 1 µL Exonuclease I (NEB) was added and incubated at 37°C for 30 min. The synthesized cDNA products were treated with 15 µL 1 M NaOH and 15 µL 0.5 M EDTA (pH 8.0) at 65°C for 15 min to digest the RNA template. The cDNA was purified with Oligo& Concentrator™ kit (Zymo research). Then 0.8 µL 80 µM 5' adaptor (Table S2) and 1 µL DMSO were added into 5 µL cDNA sample, and mixed well. The samples were heated at 75°C for 2 min and then chilled on ice immediately. 2 µL 10×RNA ligation buffer (NEB), 0.2 µL 0.1 M ATP (NEB), 9 µL 50% PEG8000 (NEB), 1.5 µL T4 RNA ligase high concentration 1 (NEB), and 0.5 µL RNase-free H_2O were added to cDNA and incubated at 25°C overnight. The cDNA was purified using DNA Clean & Concentrator™ kit (Zymo research). The amplification cycles of the cDNA were determined by qPCR (generally 10-15 cycles). The

cDNA was amplified by PCR with Q5® High-Fidelity 2×Master Mix (NEB) followed the manufacturer's protocol. Library PCR products were purified twice by 0.9×XP beads (Beckman) and then loaded to 3% agarose gel. The libraries were sequenced on Illumina NovaSeq.

Data analysis

Adapters in raw reads were trimmed, and reads shorter than 20 were filtered with Trim Galore (v0.6.1). Then reads were mapped to 60-nt and 32-nt oligonucleotides sequences with bowtie2 (v2.4.1). RT-stop rates of all bases in oligonucleotides sequences were calculated using custom scripts.

Cell culture and RNA extraction

HEK293T cells were maintained in Dulbecco's modified eagle's medium (DMEM) (high glucose) supplemented with 10% fetal bovine serum (FBS), and 1% penicillin/streptomycin (Beijing Ding-guo changsheng Biotechnology Co., Ltd) at 37°C with 5% CO_2. Total RNA was isolated from HEK293T cell lines with TRIzol according to the manufacturer's instructions (Thermo scientific). PolyA-RNA was isolated from total RNA through two rounds of poly(A)+ selection with Oligo$(dT)_{25}$ magnetic beads (NEB).

Ψ detection in total RNA using RIPS

HEK293T total RNA or mRNA fragments were performed T4 PNK treatment. 500 ng RNA fragments, 2 μL 10×T4 PNK buffer (NEB), 2 μL T4 PNK (10 U/μL, NEB), 3 μL 10 mM ATP, and 1 μL Ribolock RNase Inhibitor (40 U/μL, Thermo scientific) and RNase-free H_2O to 20 μL were incubated at 37° C for 2 h and inactivated by heating at 65℃ for 20 min. The RNA was purified using RNA Clean & Concentrator™ kit (Zymo research). CMC labeling and XRN1 treatment were the same as before with some adjustments. We used NEBuffer 1 (NEB) in XRN1 digestion steps. Reverse transcription was the same as library preparation with some adjustments. We used random hexmer (50 μM, Thermo scientific) as RT primer. HEK293T total RNA or mRNA were digested into ~100- to 150-nt fragments using RNA fragmentation solution (Thermo scientific). A portion of the RNA was as Input, the remaining half was treated with CMC only, and the other half was treated with CMC and XRN1. Two pairs of primers were designed, with one pair of primers (FP1 and RP1) located on either side of the Ψ site and the other pair (FP2 and RP2) located downstream of the Ψ site. The sequences were seen in Table S2. We used Input as control and divided the relative content of FP1 and RP1 amplification by the relative content of FP2 and RP2 amplification ($2^{-(\Delta Ct1-\Delta Ct2)}$). Stop rate was calculated using $1-2^{-(\Delta Ct1-\Delta Ct2)}$. The initial step involved the digestion of the input samples without CMC labelling to eliminate the potential influence of RNA structure and other variables on the exonuclease digestion process.

Ψ distinction in RNA single-stranded and double-stranded region

Two complementary RNA strands were annealed to form a double-stranded structure with a procedure of heating at 95° C for 5 min, then gradually cooled to 20° C with a rate of -1° C/min, lastly heating at 20℃ for 5 min. The double-stranded RNA formation was observed by natural polyacrylamide gel electrophoresis. These single-stranded RNA and double-stranded RNA were reacted with 0.2 M CMC in borate buffer (50 mM $Na_2B_4O_7$ pH 8.0, 100 mM KCl, 5 mM $MgCl_2$) at 37°C for 30 min followed by RNA purification with ethanol precipitation or kit. The alkaline treatment was the same as above and RNA was purified. The products were analyzed on a urea-PAGE. The 60-RNA-Ψ annealing step was the same as above. Single-stranded or double-stranded RNA and their mixture were treated with CMC, alkaline and XRN1 as before. The enzymatic cleavage was studied by denaturing gel electrophoresis, and then the samples were performed library construction as above.

Supplementary Tables and Figures

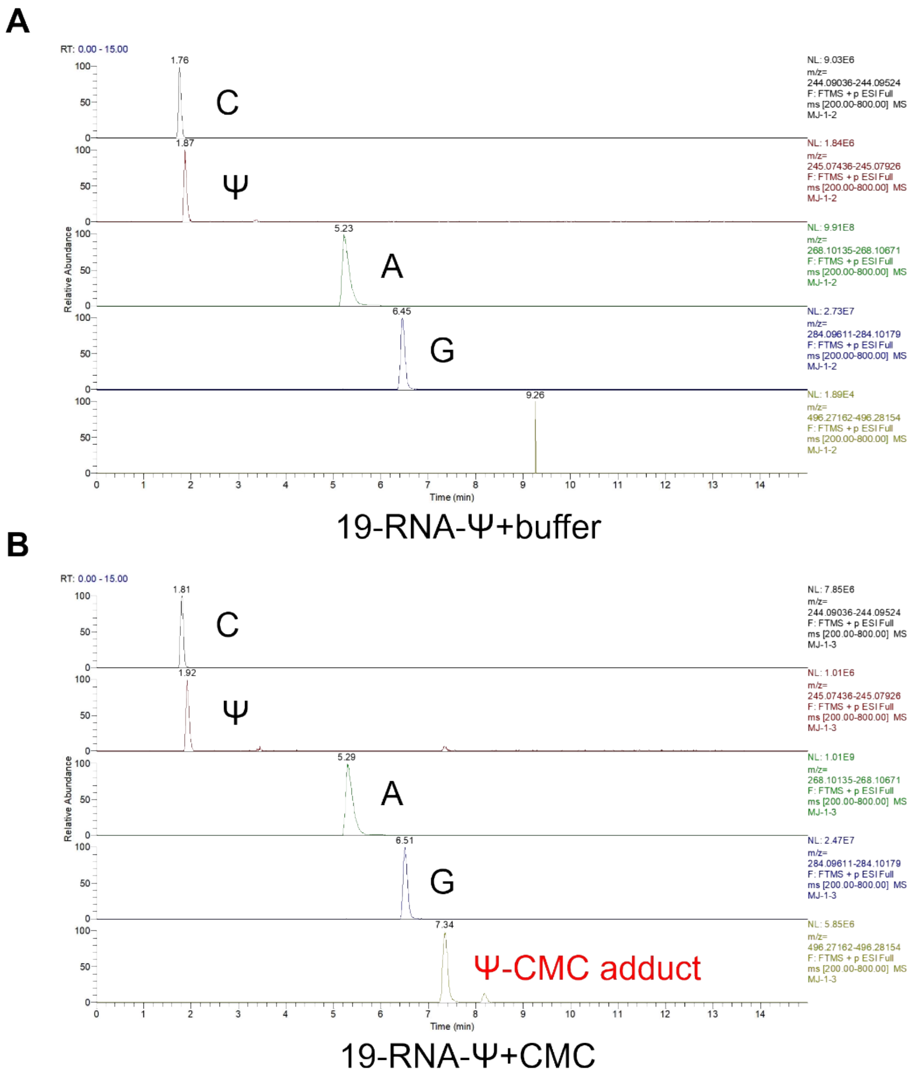

Figure S1. Validation of the Ψ sites specifically labeled with CMC by HPLC-MS/MS chromatograms. (A) RNA samples that incubated with buffer and treated with alkaline were performed enzymatic digestion and separation by HPLC-MS/MS. (B) RNA samples that incubated with CMC and treated with alkaline were performed enzymatic digestion and separation by HPLC-MS/MS.

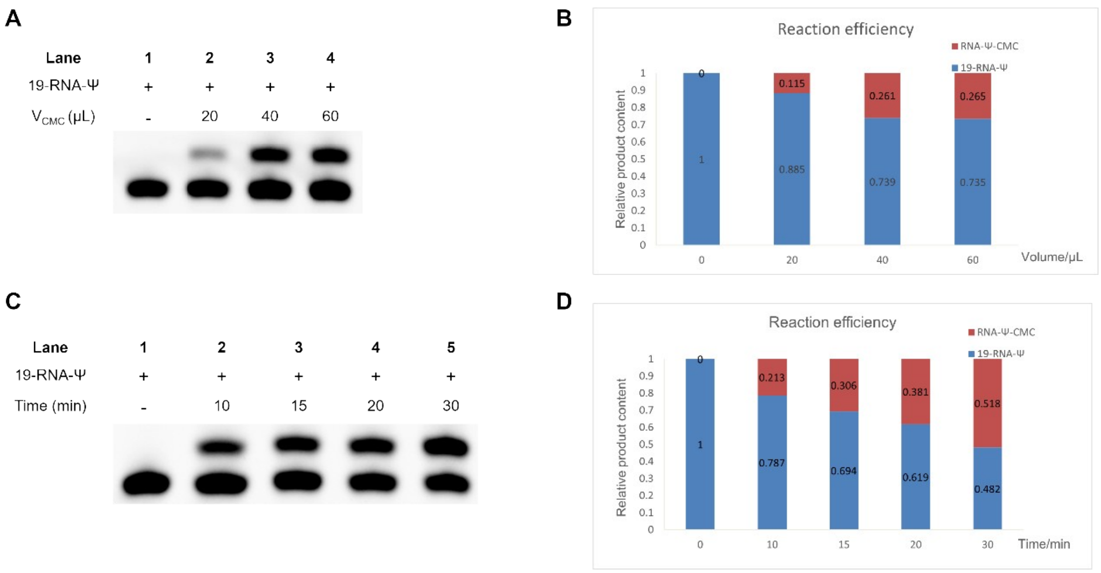

Figure S2. Control of experimental conditions for CMC-specific labeling of Ψ. (A) Optimization of CMC concentration. (B) Relative content of RNA-Ψ-CMC. The relative quantities were calculated by comparing signal of raw materials and products. (C) Optimization of reaction time. (D) Relative content of RNA-Ψ-CMC. The relative quantities were calculated by comparing signal of raw materials and products.

	Calculated	Found
16-RNA-Ψ	5855.7	5856.2
16-RNA-Ψ-CMC+XRN1	6107.9	6107.8

	Calculated	Found
19-RNA-Ψ	6835.3	6835.3
19-RNA-Ψ-CMC+XRN1	6107.9	6107.4

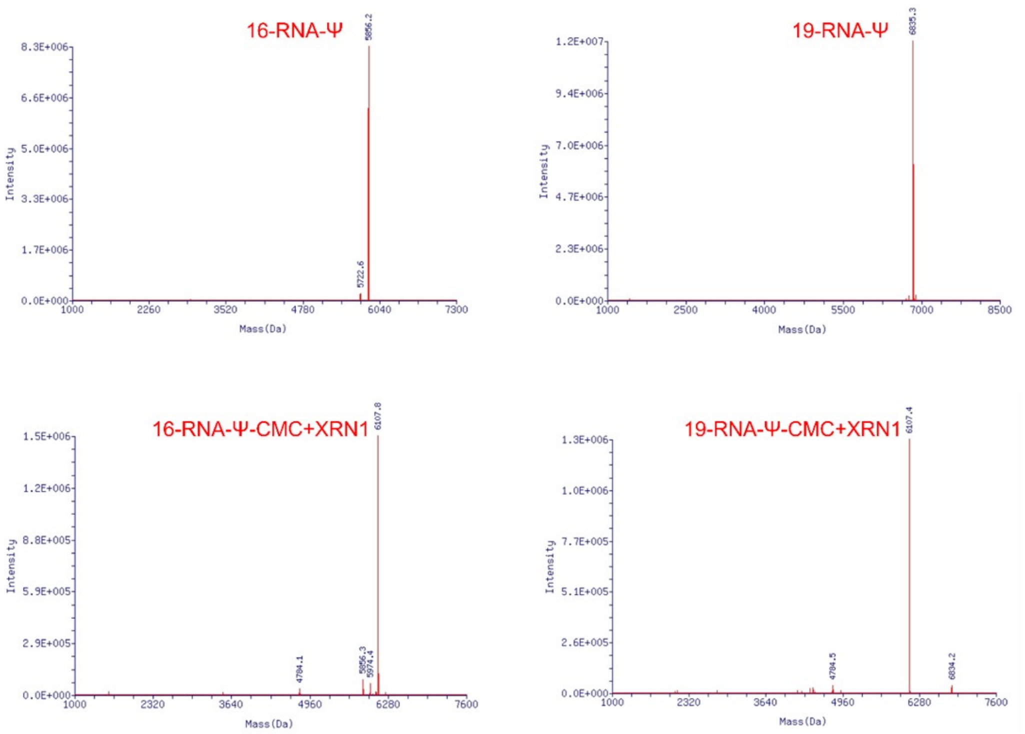

Figure S3. ESI-MS analysis of 16-RNA-Ψ, 19-RNA-Ψ and their corresponding products treated with both CMC and XRN1.

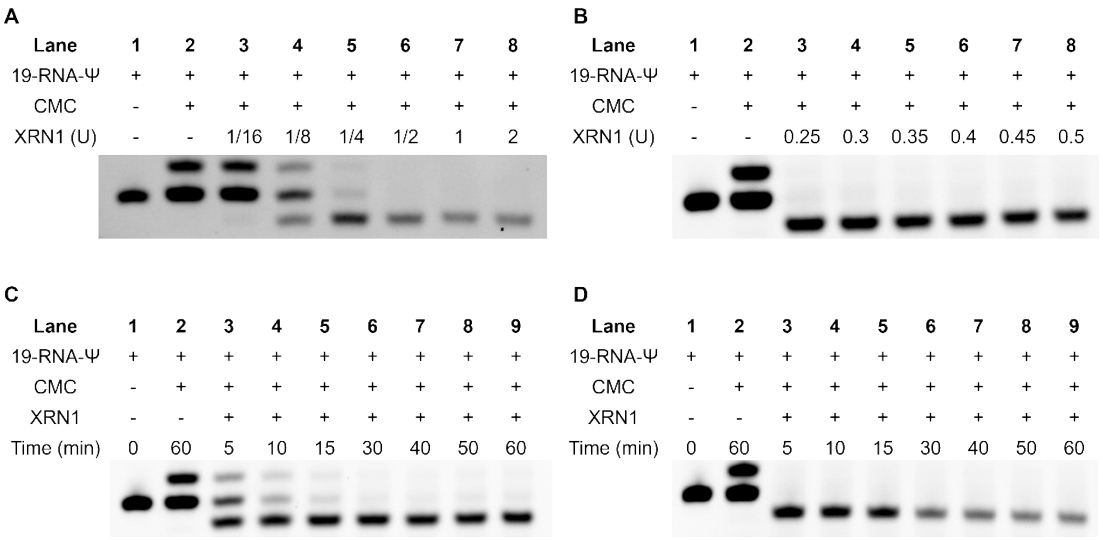

Figure S4. Optimization of experimental conditions for RIPS strategy. (A and B) Optimization of XRN1 enzyme concentration conditions with a fixed digestion time of 1 h. (C and D) Optimization of XRN1 digestion time. (C) XRN1 with 0.25 U was used for the enzyme digestion time gradient. 5, 10, 15, 30, 40, 50, and 60 min time gradients were selected. (D) XRN1 with 0.5 U was used for the enzyme digestion time gradient. 5, 10, 15, 30, 40, 50, and 60 min time gradients were selected.

A

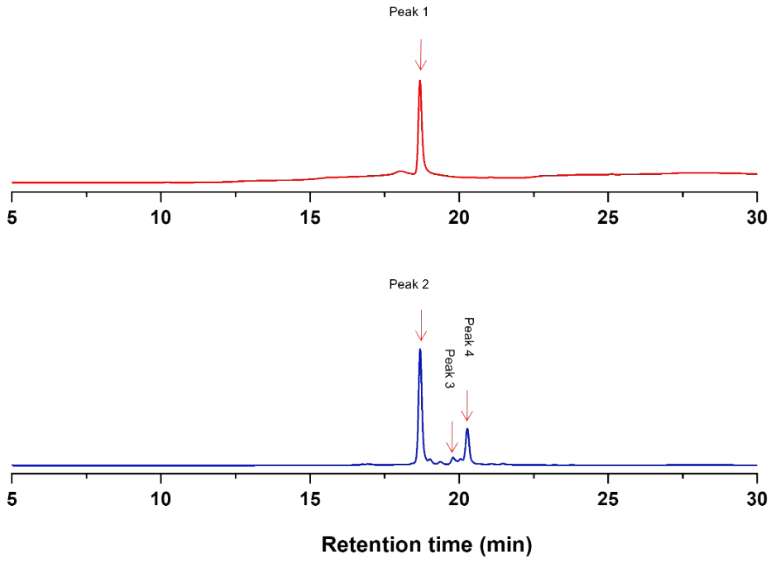

B

Lane	1	2	3	4	5	6
Peak	-	-	1	2	3	4
19-RNA-Ψ	+	+	+	+	+	-
RNA-Ψ-CMC	-	+	-	-	+	+
Retention time (min)	-	-	18.332	18.488	19.8	20.379

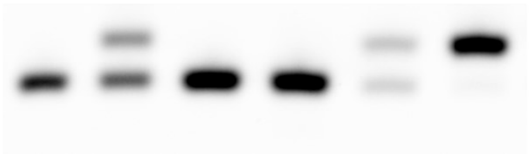

Figure S5. Isolation and identification of RNA-Ψ-CMC. (A) 19 nt RNA was incubated with CMC and RNA-Ψ-CMC was isolated by HPLC. The top was the raw material, and the bottom was the sample after the reaction with CMC. The peaks were marked on the graph. (B) Gel electrophoresis analysis of recovered products for each peak of HPLC.

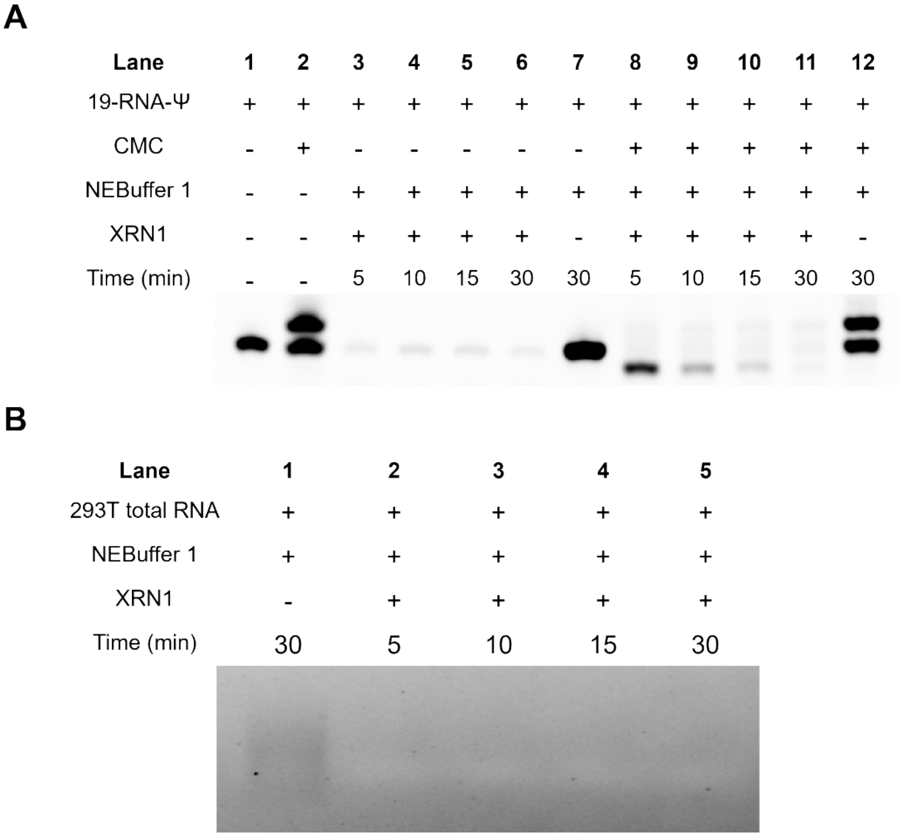

Figure S6. Digestion situation using NEBuffer 1 with RIPS. (A) 19 nt RNA treated with or without CMC were digested using XRN1 with time gradient. 5, 10, 15, and 30 min time gradients were selected. (B) Fragmented HEK293T total RNA were digested using XRN1 with time gradient. 5, 10, 15, and 30 min time gradients were selected.

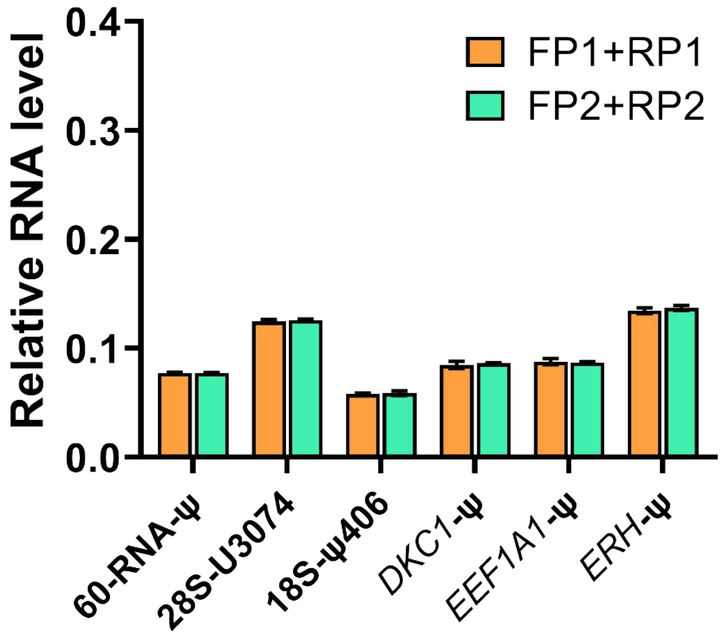

Figure S7. Relative RNA level of Input samples after XRN1 digestion were analyzed by qPCR.

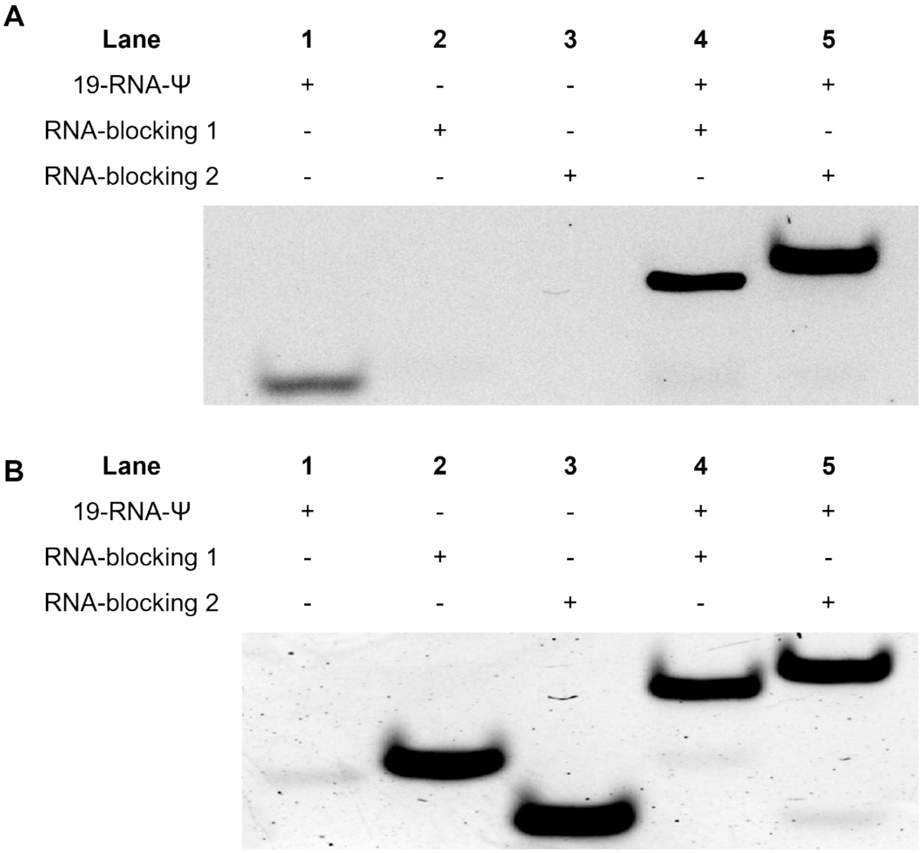

Figure S8. Annealing results of complementary RNA. (A) The RNA was visualized by the fluorecence of FAM. (B) The RNA was visualized by GelRed.

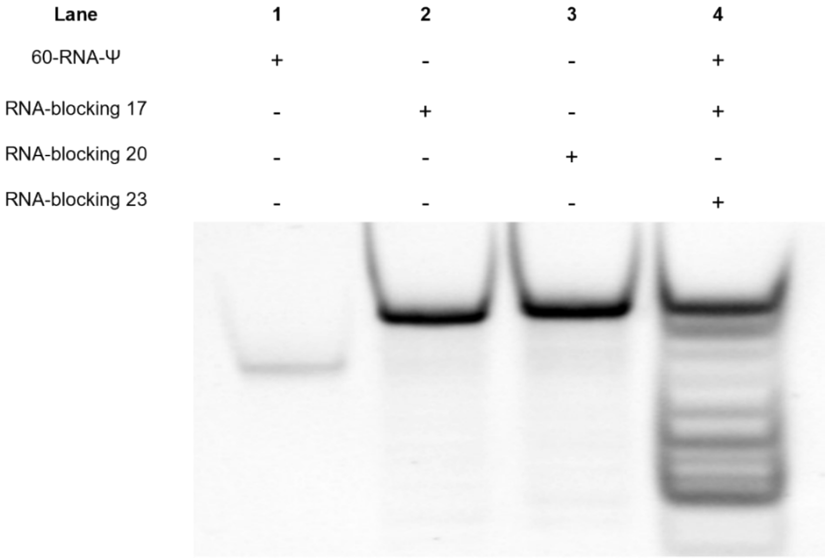

Figure S9. Annealing results of 60 nt RNA and its complementary different lengths of RNA-blocking chains. The RNA was visualized by GelRed.

Table S1. Name and sequence of the RNA used in the experiment.

Name	Sequence
16-RNA-Ψ	5'-(PO_4) ΨGAGACGCGAACAGGA(FAM)-3'
19-RNA-Ψ	5'-(PO_4)GCAΨGAGACGCGAACAGGA(FAM)-3'
19-RNA-U	5'-(PO_4)GCAUGAGACGCGAACAGGA(FAM)-3'
60-RNA-Ψ	5'-(PO_4)CAGCACACGAAAACCAAGAGΨACGCCGAAAGAGACACGCACGAACGAGACAGAACAGGAC-3'
RNA-blocking 1	5'-UCCUGUUAUCGUCUCAUGC-3'
RNA-blocking 2	5'-GACUGUGCGCGUCUCAUGC-3'
RNA-blocking 17	5'-UCGGCGUACUCUUGGUU-3'
RNA-blocking 20	5'-UCGGCGUACUCUUGGUUUUC-3'
RNA-blocking 23	5'-CUUUCGGCGUACUCUUGGUUUUC-3'
32-RNA-Ψ	5'-(PO_4)GCACUACΨAUCUAUGAAUCUCGAAUGUGAAGG-3'

Table S2. Name and sequence of the primer used in the experiment.

Primer	Sequence
32-RNA-Ψ RT primer	5'-ACACGACGCTCTTCCGATCTCCTTCACATTCGAGATTCAT-3'
60-RNA-Ψ RT primer	5'-ACACGACGCTCTTCCGATCTGTCCTGTTCTGTCTCGTTCG-3'
5' adaptor	5'-(PO_4) NNNNNNNNNNAGATCGGAAGAGCACACGTCTG(3SpC)-3'
60-RNA-Ψ qPCR FP1	5'-CAGCACACGAAAACCAAGAG-3'
60-RNA-Ψ qPCR RP1	5'-GTCCTGTTCTGTCTCGTTCG-3'
60-RNA-Ψ qPCR FP2	5'-ACGCCGAAAGAGACACGC-3'
60-RNA-Ψ qPCR RP2	5'-GTCCTGTTCTGTCTCGTTC-3'
28S-U3074 qPCR FP1	5'-CTCTCTCCCCCGCTCCCCGT-3'
28S-U3074 qPCR RP1	5'-CGCCCCCACGCGGCGCTC-3'
28S-U3074 qPCR FP2	5'-GGGAGCGCCGCGTGGGGG-3'
28S-U3074 qPCR RP2	5'-GCCGCCCCGACCCTTCTCCC-3'
18S-Ψ406 qPCR FP1	5'-TTTCGATGGTAGTCGCCGTG-3'
18S-Ψ406 qPCR RP1	5'-CTCAGGCTCCCTCTCCGGAA-3'
18S-Ψ406 qPCR FP2	5'-GAATCAGGGTTCGATTCCGG-3'
18S-Ψ406 qPCR RP2	5'-TCCTTGGATGTGGTAGCCGT-3'
DKC1-Ψ qPCR FP1	5'-ATAAGCTGAATGTAAGGACAACACAC-3'
DKC1-Ψ qPCR RP1	5'-ACCTGTCCTGATATAGTCCCCAATC-3'
DKC1-Ψ qPCR FP2	5'-GAGATTGGGGACTATATCAGGACAGG-3'
DKC1-Ψ qPCR RP2	5'-CCTCATGGGAAGAGGGGTTAGAGG-3'
EEF1A1-Ψ qPCR FP1	5'-TTGGTGGTATTGGTACTGTTCCTGT-3'
EEF1A1-Ψ qPCR RP1	5'-ACGTTGACTGGAGCAAAGGTGA-3'
EEF1A1-Ψ qPCR FP2	5'-AATGGATTCCACTGAGCCAC-3'
EEF1A1-Ψ qPCR RP2	5'-CTGACTTCCTTAACAATTTCCTCATATC-3'
ERH-Ψ qPCR FP1	5'-TGATGATTTAGATGGAAGTTGTTCTTCG-3'
ERH-Ψ qPCR RP1	5'-GGGTTAGTATGTGAATGTTATAAAGTAGAT-3'
ERH-Ψ qPCR FP2	5'-CATATCTACTTTATAACATTCACATACTAACCC-3'
ERH-Ψ qPCR RP2	5'-CCTCATGGGAAGAGGGGTTAGAGG-3'

Table S3. Name and specific site of the RNA detected in the experiment.

Gene name	Accession number	Site
DKC1	NR_110022.2	327
EEF1A1	NM_001402	875
ERH	NM_004450.3	649

References

1. Li, X. Y.; Zhu, P.; Ma, S. Q.; Song, J. H.; Bai, J. Y.; Sun, F. F.; Yi, C. Q., Chemical pulldown reveals dynamic pseudouridylation of the mammalian transcriptome. *Nature Chemical Biology,* **2015,** *11* (8), 592-597.

2. Bakin, A. V.; Ofengand, J., Mapping of pseudouridine residues in RNA to nucleotide resolution. *Methods in molecular biology (Clifton, N.J.),* **1998,** *77*, 297-309.

3. Koh, C. W. Q.; Goh, Y. T.; Goh, W. S. S., Atlas of quantitative single-base-resolution N-6-methyl-adenine methylomes. *Nature Communications,* **2019,** *10*, 5636.
